# Supplementary figures and images for: Anticancer Activity of Delta-Tocotrienol in Human Hepatocarcinoma: Involvement of Autophagy Induction
Source: Cancers (Basel). 2024 Jul 26;16(15):2654. doi: 10.3390/cancers16152654 (PMC11311296; doi:10.3390/cancers16152654)

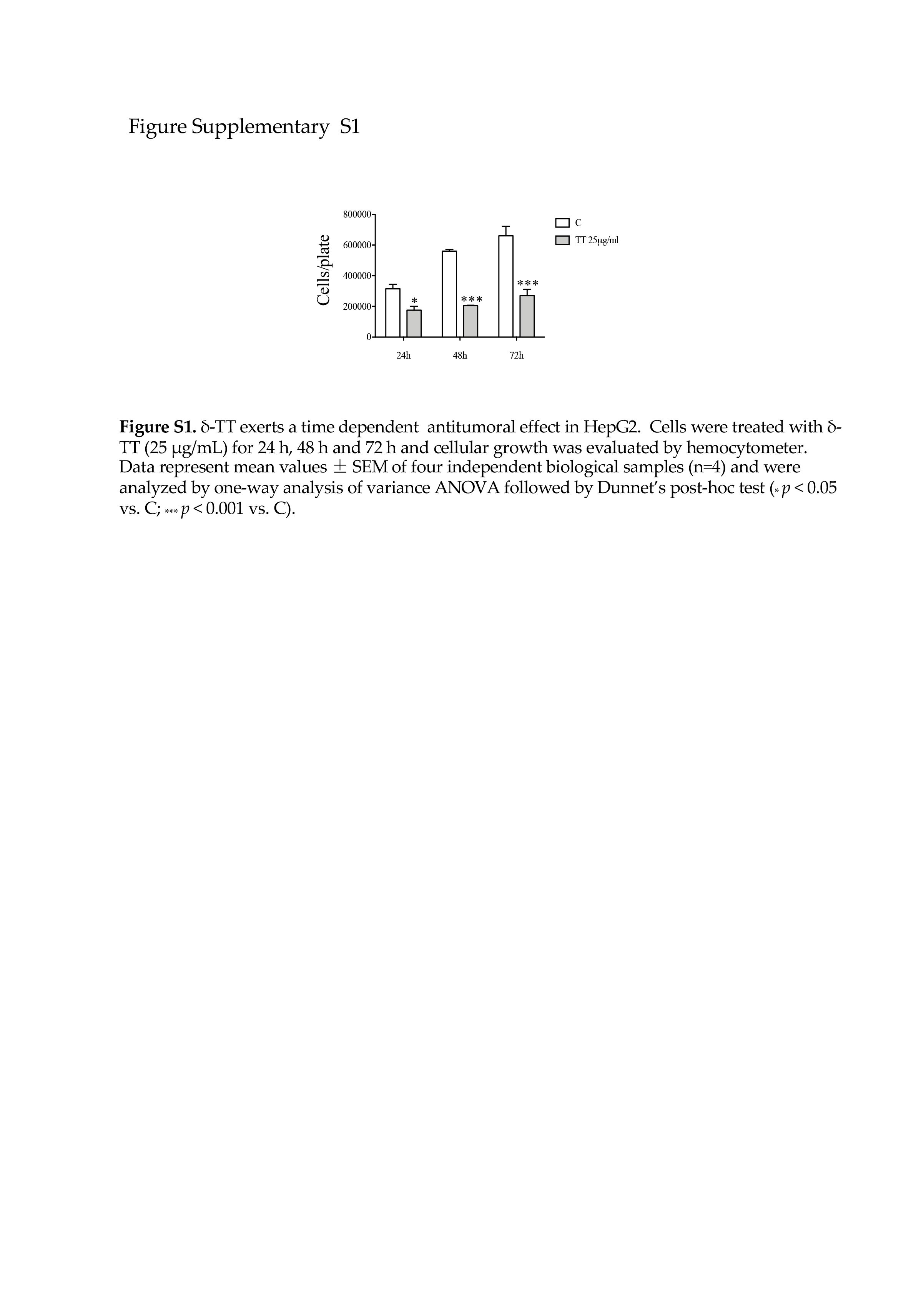

Supplement: Supplementary file 1 [file cancers-16-02654-s001.zip › cancers-3077449-Figure S1.tif]

Figure 1

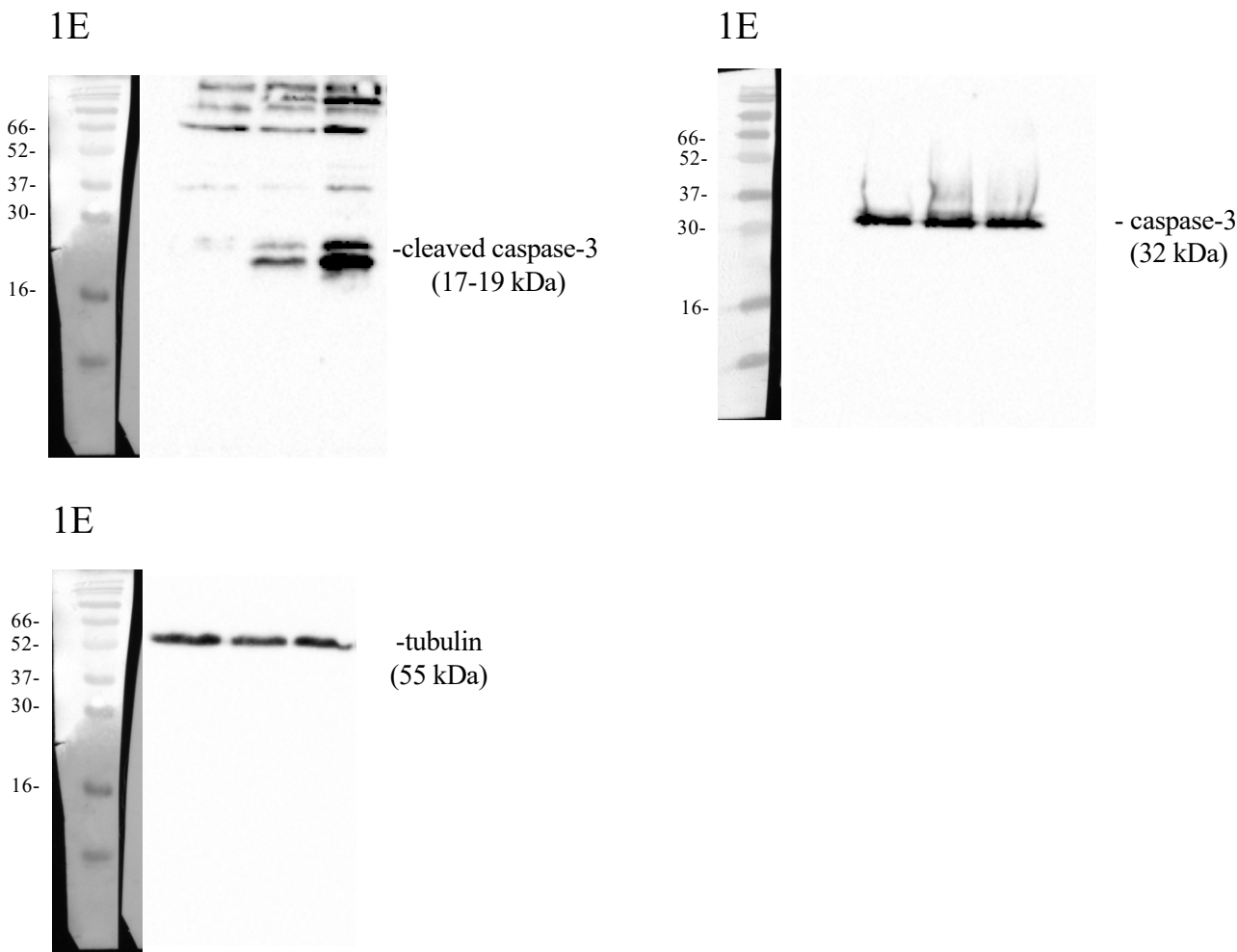

Figure 3

3B

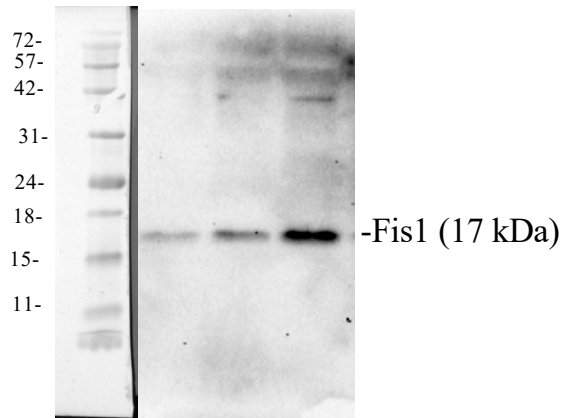

3B

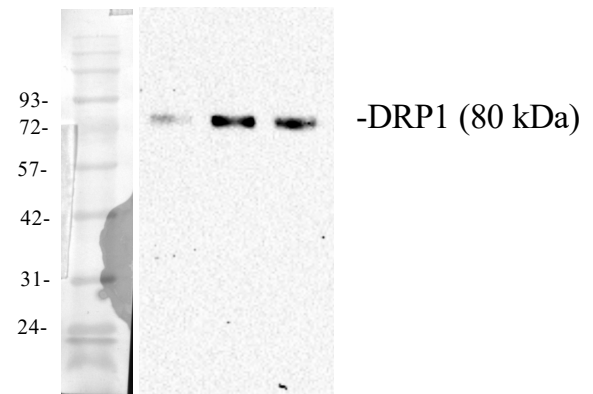

3B

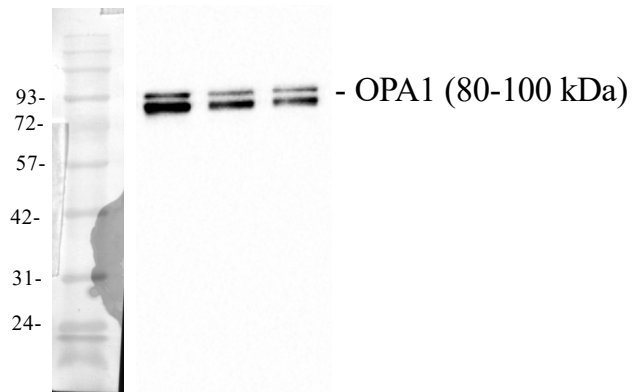

3B

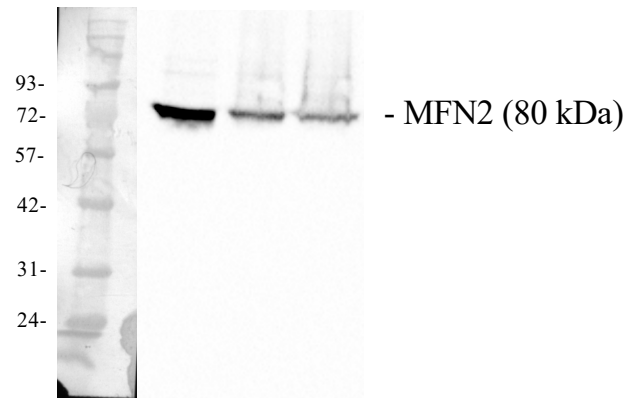

3B

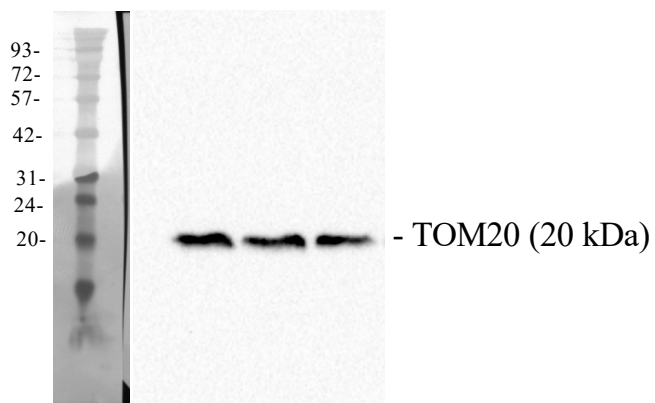

3B

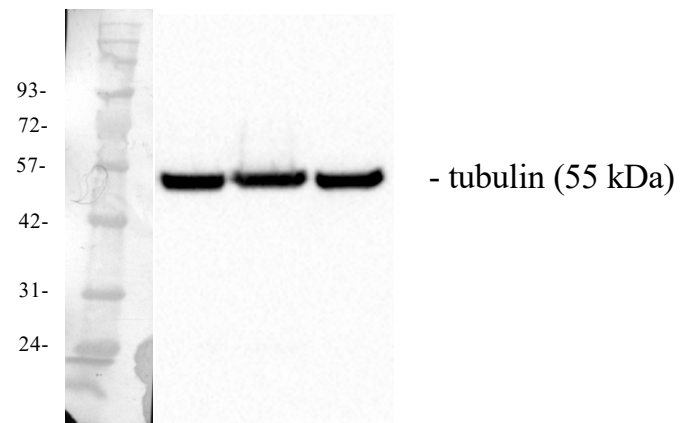

Figure 4

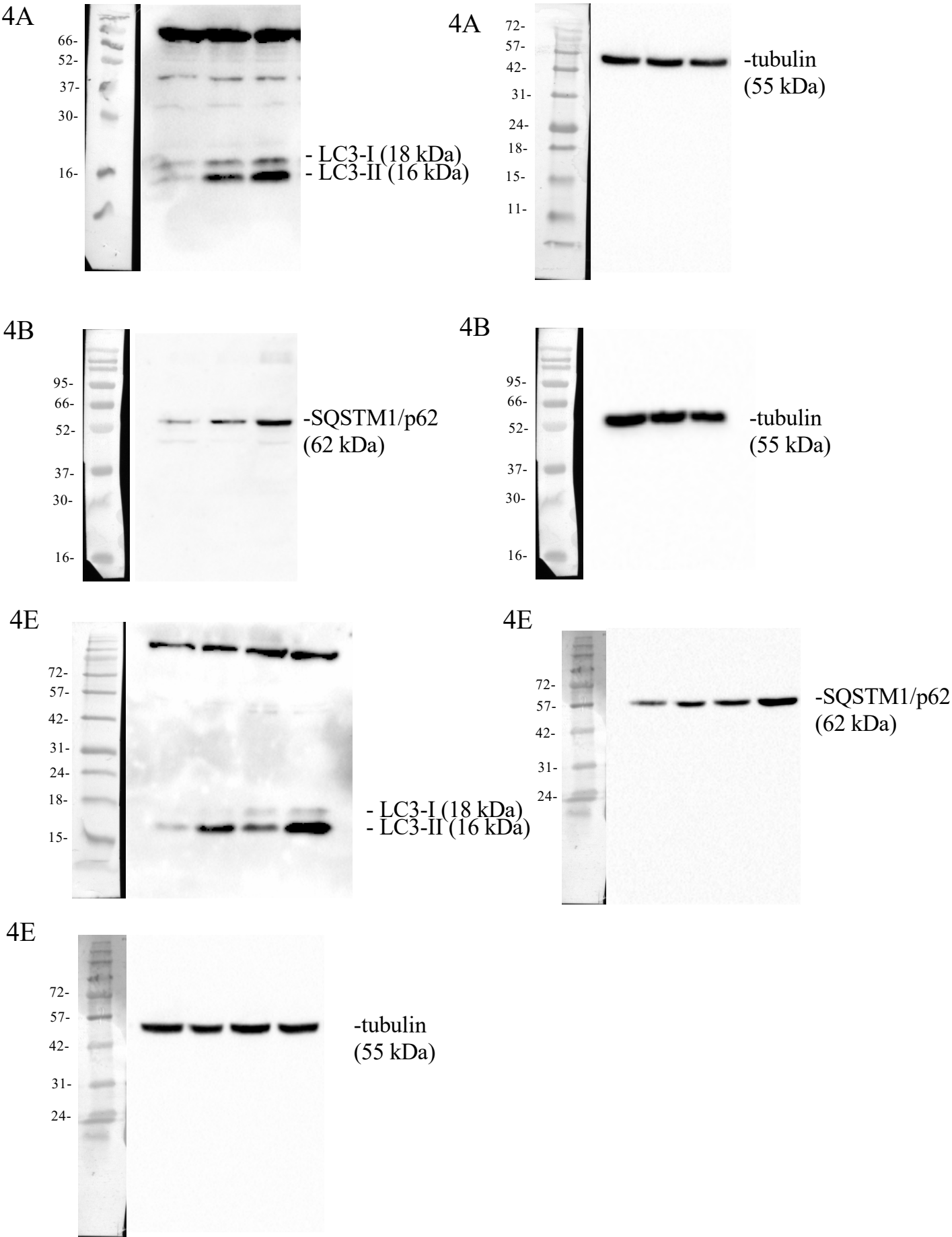

Figure 5

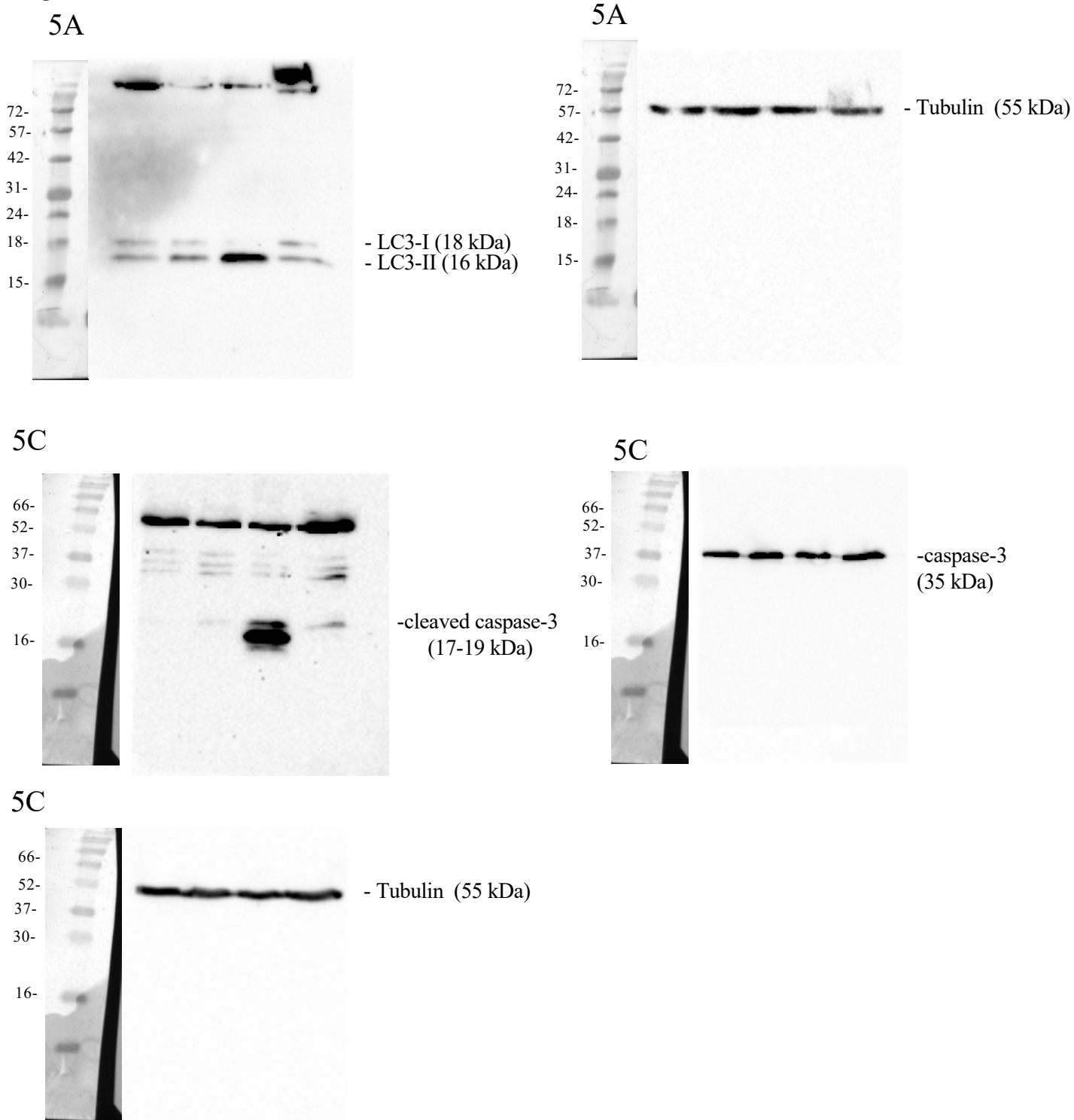

Figure 5

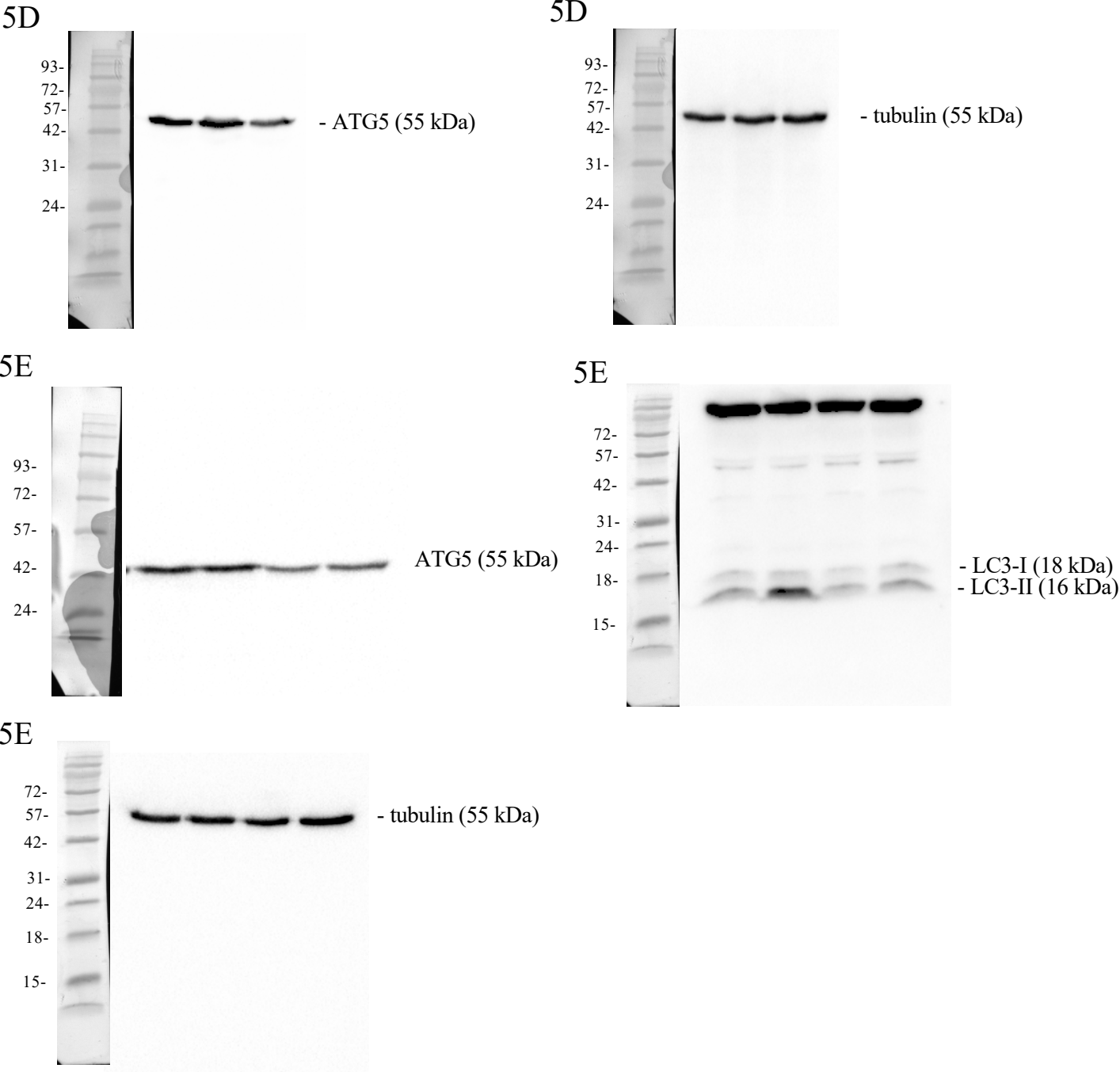

Figure 5

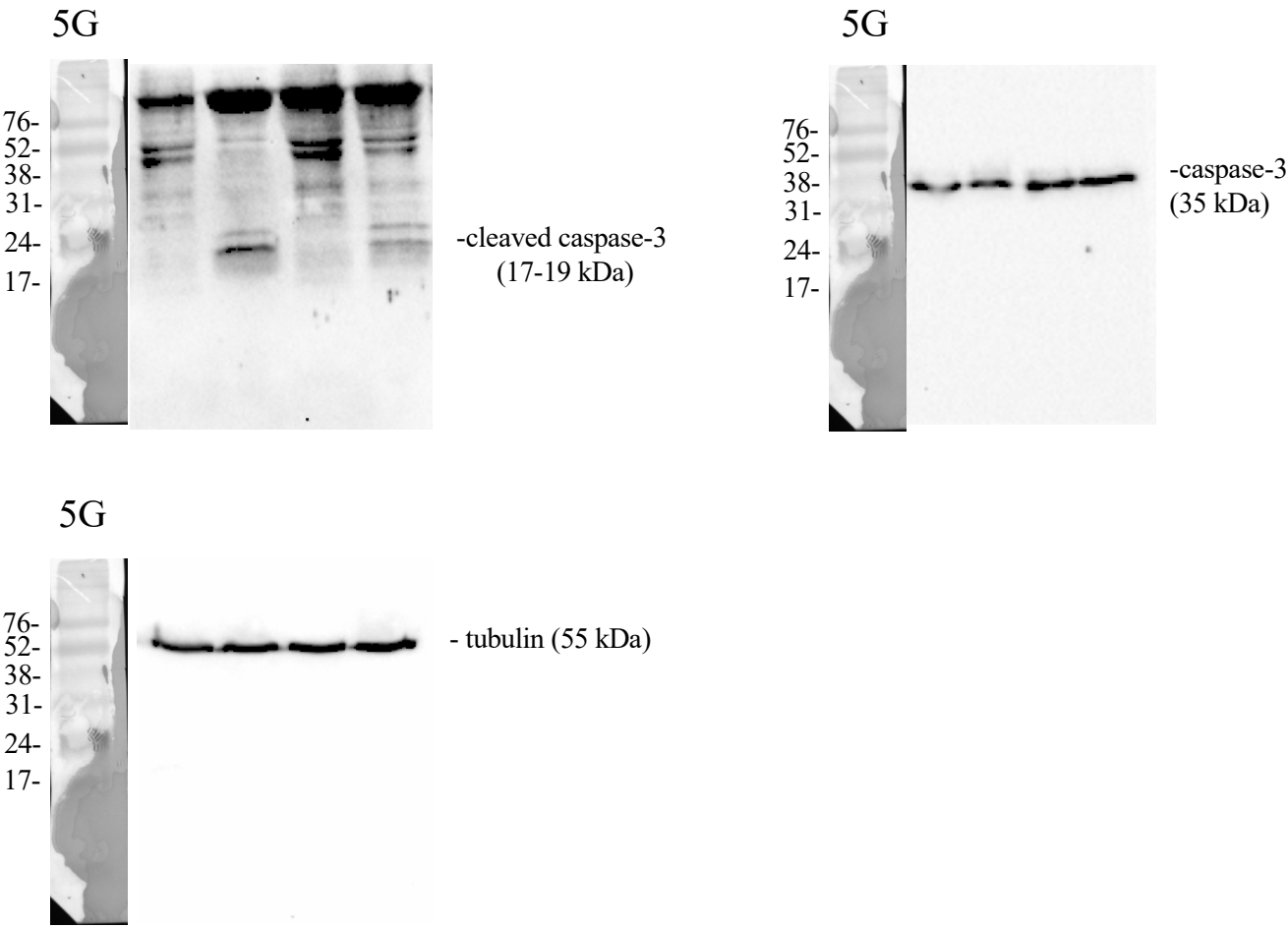

Figure 6

6B

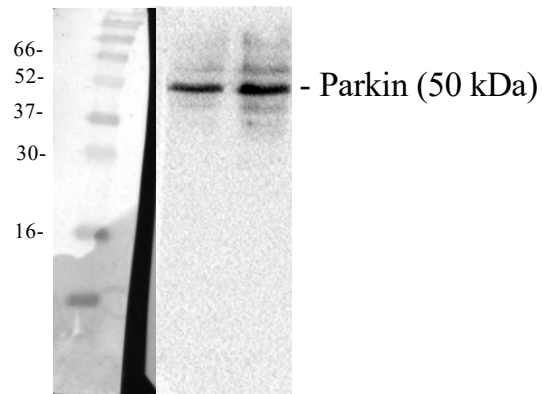

6B

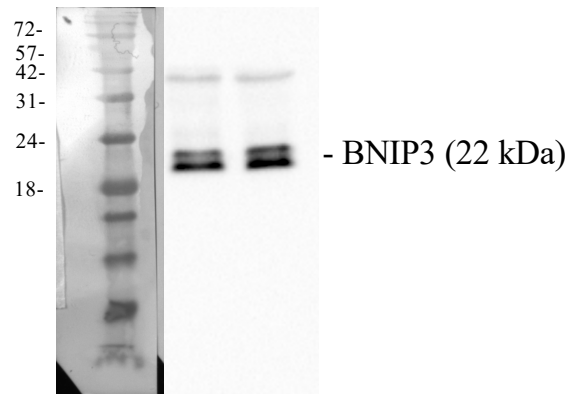

6B

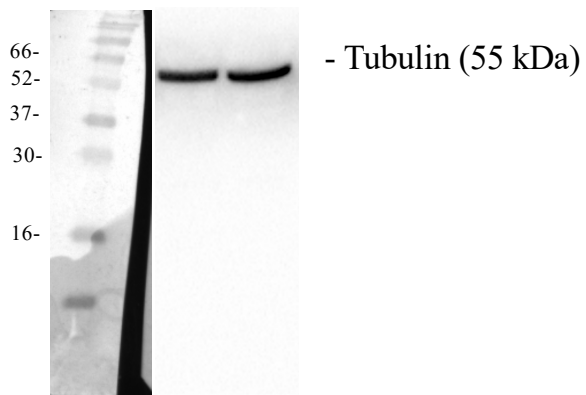

6C

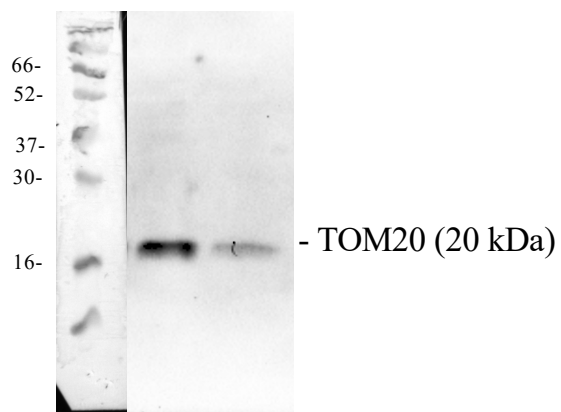

6C

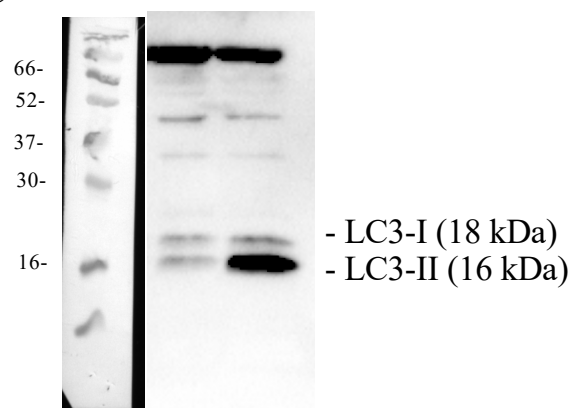

6C

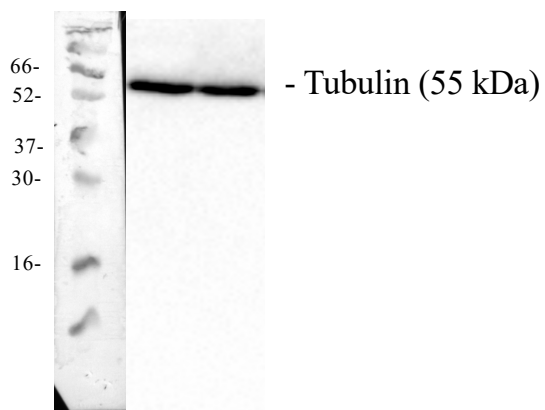

6F

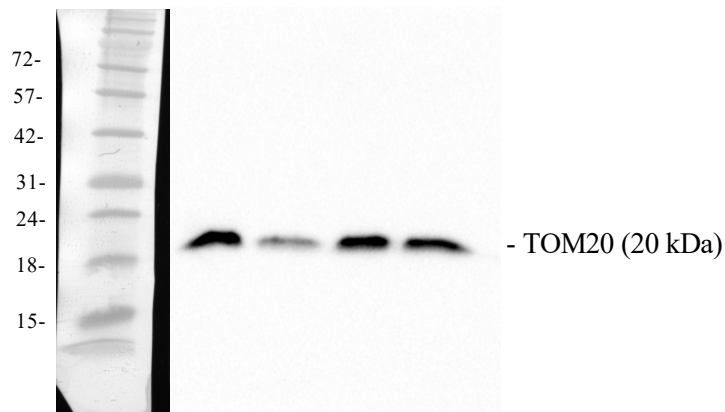

6F

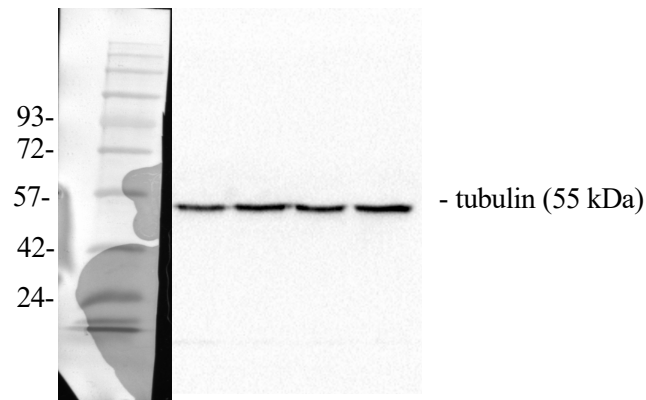

Supplement: Supplementary file 1 [file cancers-16-02654-s001.zip › cancers-3077449-File S1.pdf]
